# Toxigenic *Aspergillus* Diversity and Mycotoxins in Organic Spanish Grape Berries

**DOI:** 10.3390/toxins17100487

**Published:** 2025-09-30

**Authors:** Clara Melguizo, Andrea Tarazona, Jéssica Gil-Serna, Fernando Mateo, Belén Patiño, Eva María Mateo

**Affiliations:** 1 Department of Genetics, Phisiology and Microbiology, Faculty of Biological Sciences, University Complutense of Madrid, 28040 Madrid, Spain; claramel@ucm.es (C.M.); jgilsern@ucm.es (J.G.-S.); 2 Department of Microbiology and Ecology, Faculty of Biology, University of Valencia, 46100 Valencia, Spain; andrea.tarazona@uv.es; 3 Department of Electronic Engineering, School of Engineering (ETSE), University of Valencia, 46100 Valencia, Spain; fernando.mateo@uv.es; 4 Department of Microbiology and Ecology, Faculty of Medicine, University of Valencia, 46010 Valencia, Spain

**Keywords:** organic grapes, toxigenic fungi, mycotoxins, *Aspergillus*, ochratoxin A, aflatoxin B_1_, fumonisin B_2_

## Abstract

Grapes are frequently contaminated by *Aspergillus* section *Nigri* fungi and ochratoxin A (OTA), with *A. niger* also capable of producing substantial fumonisin B_2_ (FB_2_) levels. Emerging evidence suggests that aflatoxigenic fungi may eventually replace ochratoxigenic fungi in certain regions due to better adaptation to changes in climatic conditions. However, research on the toxigenic fungal community and mycotoxins in grapes from organic vineyards remains limited. Research on Spanish conventional grapes is also deficient, with most of the available literature being outdated. The present study investigates the diversity of toxigenic fungi and the presence of mycotoxins in organically cultivated grape berries in Spain, which are renowned for their significant oenological tradition. This study employed species-specific PCR protocols for fungal characterization and optimized methods for the analysis of OTA, FB_2_, and aflatoxin B_1_ (AFB_1_) by UPLC–ESI–MS/MS. The most prevalent species present were *Aspergillus flavus*, *A. niger*, *A. parasiticus*, *A. steynii*, *A. carbonarius*, and *A. westerdijkiae* (67.1%, 43.5%, 20.0%, 14.1%, 14.1%, and 11.8% of the samples, respectively). OTA was detected only in 16 samples (19%), averaging 0.48 ng/g and peaking at 0.7 ng/g, which were lower than previously reported for conventional grapes. There was no FB_2_ or AFB_1_ detected. This study is pioneering in its exploration of the occurrence of toxigenic mycobiota, beyond *Nigri* fungi, and subsequent potential for other serious mycotoxins to contaminate Spain’s organic grapes.

## 1. Introduction

According to a 2023 report by the International Organization of Vine and Wine (OIV), vineyards cover approximately 7.2 million hectares globally, underscoring their significant role in agriculture [1]. Economically, the wine export market achieved a value of 36.0 billion euros in 2023 [2]. Spain stands out as the leading country for vineyard surface area, with around 945,000 hectares dedicated to grape cultivation. Grapes are the fourth most harvested crop in Spain, following olives, barley, and wheat [3].

A major challenge in viticulture is the contamination of grapes by mycotoxins, which are harmful secondary metabolites produced by certain filamentous fungi. These contaminants not only reduce grape quality and yield but also pose serious health risks to both humans and animals. As a result, the European Union (EU) has established strict regulations to control mycotoxin levels in food and feed [4].

Among mycotoxins affecting grapes, ochratoxin A (OTA) is the most common contaminant. Fungal species such as *Aspergillus carbonarius* and *A. niger* (i.e., the black aspergilli) are the primary producers of OTA and frequently contaminate vineyards. Other toxigenic species, from sections *Flavi* (*A. flavus*, *A. parasiticus*) and *Circumdati* (*A. westerdijkiae*, *A. steynii*), have also been detected in vineyards, although at lower levels [5,6]. Because of the health issues linked to OTA nephrotoxicity [7], *A. carbonarius* poses greater economic and health threats due to its substantial OTA-producing ability, despite being less prevalent than *A. niger* [5,8,9,10].

Beyond OTA, some *A. niger* strains can also produce fumonisins, such as fumonisin B_2_ (FB_2_), the predominant fumonisin associated with this species [11,12,13,14]. This mycotoxin has been shown to have a strong cytotoxic effect against several cell lines, such as hepatocytes or gastrointestinal cells [15,16]. The FB_2_ toxin has been detected in grapes, must, and wine [17,18,19]. Another serious mycotoxin, aflatoxin B_1_ (AFB_1_), mainly produced by *A. flavus* and *A. parasiticus* [20] and recognized as the most potent known natural hepatocarcinogen [21,22], has been found in these products, although studies on its occurrence remain limited [23,24]. Despite these findings, OTA continues to be the primary mycotoxin of concern in grapes. However, recent studies suggest that AFB_1_ could become a greater threat in certain European regions due to shifts in climatic conditions, particularly rising temperatures that favor the growth of aflatoxigenic fungi [25]. Paterson et al. [6] emphasized the importance of monitoring AFB_1_ production in vineyards, as warmer and drier weather conditions may favor the growth and virulence of aflatoxigenic fungi over ochratoxigenic fungi.

To reduce the risks associated with OTA, the EU has set maximum allowable levels for this mycotoxin in grape products: 2 μg/kg (parts per billion) in wine and grape juice, and 8 μg/kg in dried grape fruits such as raisins and sultanas [4]. Currently, there are no established limits for acceptable levels of fumonisins and aflatoxins in grapes or wine.

Despite the growing relevance of this issue, studies on fungal diversity in vineyards, especially under changing climate scenarios, remain limited. Most existing studies focus on conventionally managed vineyards, with little information available regarding the occurrence of toxigenic species in organic vineyards. Organic agriculture has grown significantly over the last few decades, driven by increased consumer awareness of health and environmental sustainability. In 2022, organic farming covered 16.9 million hectares across Europe, representing about 10.5% of the total utilized agricultural area [26]. The European Commission’s Farm to Fork Strategy aims to increase this percentage to 25% by 2030 [27]. Spain is the second-largest producer of organic products in Europe and ranks seventh globally, with 2.6 million hectares devoted to organic farming [28]. Thus, 13% of European organic farmland is dedicated to permanent crops such as grapes, accounting for approximately 0.5 million hectares, and Spain devotes the largest area (166.285 hectares) to its organic vineyards [28].

Organic farming avoids synthetic agrochemicals, relying instead on biological control methods and natural fertilizers, such as composted plant materials and animal manure [29,30]. Additionally, cultivation techniques, including defoliation, pruning, and irrigation, are employed to optimize yield and crop quality [29]. Studies have indicated that microbial diversity is strongly influenced by agricultural management practices [31]. Nevertheless, only a few investigations have explored how organic farming affects the distribution of toxigenic fungi in vineyards. Most grape infection research focuses on *Aspergillus* section *Nigri*, often excluding other potentially mycotoxigenic species by metataxonomic approaches that lack the resolution to accurately distinguish between *Aspergillus* species [32,33].

To the best of our knowledge, no prior research has explored the occurrence of toxigenic fungi and their associated mycotoxins in Spanish organic vineyards. Moreover, existing studies in conventional vineyards throughout Spain are limited in both scope and duration [5,8]. Therefore, this study aims to address these knowledge gaps by applying targeted PCR protocols to investigate the occurrence of important toxigenic fungi in organic Spanish vineyards, as well as using optimized ultra-performance liquid chromatography–electrospray ionization–tandem mass spectrometry (UPLC–ESI–MS/MS) methods to detect the presence of OTA, AFB_1_, and FB_2_ in organic grapes.

## 2. Results

### 2.1. Occurrence of Toxigenic Aspergillus in Organic Grape Samples

**Figure 1:**
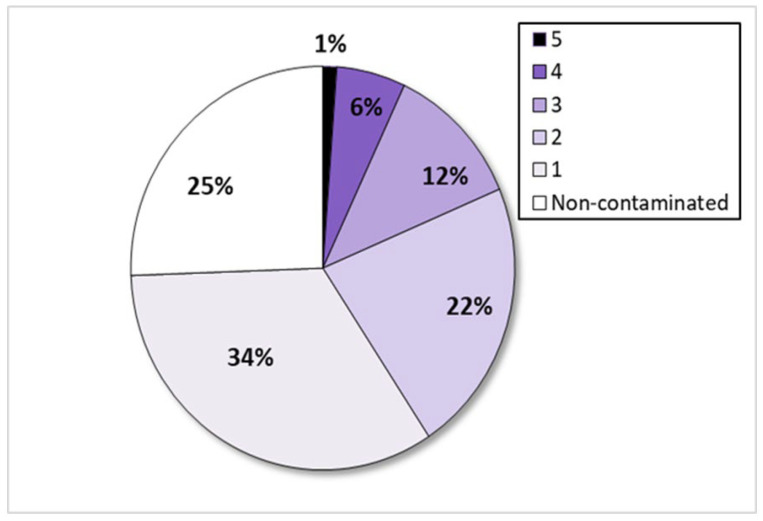
Pie graph showing the percent distribution of samples based on the number of different toxigenic *Aspergillus* species (0 to 5) present within them.

Among 85 samples tested, 75% were contaminated with at least one of the six *Aspergillus* species of interest. Additionally, 41% of the samples contained two or more toxigenic fungi. None of the samples was contaminated with all six species (Figure 1). The presence of toxigenic *Aspergillus* spp. in the analyzed samples is shown in Table S1.

**Figure 2:**
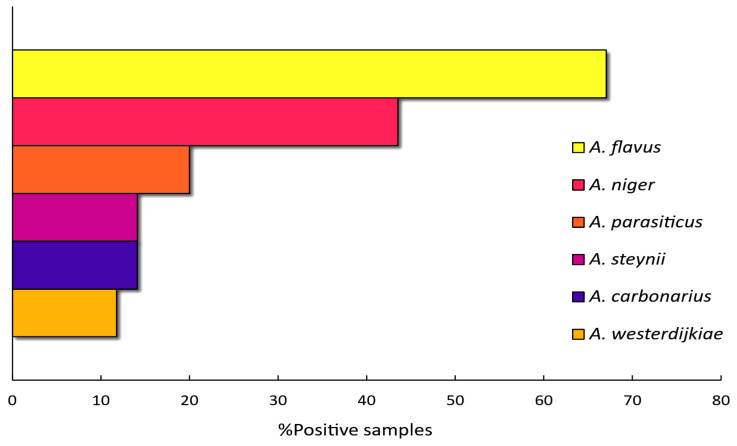
Bar graph showing percentage of samples found to contain each toxigenic *Aspergillus* species, relative to the total number of samples analyzed.

*Aspergillus flavus* was the most frequently detected species, present in 67.1% of the samples, followed by *A. niger* (43.5%) and *A. parasiticus* (20%). Both *A. steynii* and *A. carbonarius* were detected in 14.1% of the samples, while *A. westerdijkiae* was the least frequently detected species, only found in 11.8% of the grape samples (Figure 2).

### 2.2. Mycotoxin Analysis

#### 2.2.1. Determining the Best Extraction Method for UPLC Analysis of OTA

Three different extraction methods were assessed for their ability to facilitate the highest quality of UPLC analysis of OTA: dispersive liquid–liquid microextraction (DLLME), liquid–liquid extraction (LLE), and quick, easy, cheap, effective, rugged, and safe (QuEChERS) extraction. The limits of detection (LOD) for OTA using DLLME, LLE, and QuEChERS were 0.05, 0.32, and 0.2 ng/g, respectively, while the corresponding limits of quantification (LOQ) were 0.15, 0.94, and 0.6 ng/g. Preliminary assays using the three methods for OTA showed that the DLLME method achieved higher OTA recoveries from fortified blank grape samples compared to the other two techniques (Table 1). As a result, OTA analyses from our 85 samples were conducted using the DLLME method.

#### 2.2.2. Mycotoxin Detection in Grape Samples

Table 2 shows the average recoveries and the relative standard deviation (RSD) of recoveries for FB_2_ and AFB_1_, as well as their LOD and LOQ values obtained using the respective analytical methods. The mean recovery of FB_2_ was 79%, and the mean RSD was 10.6%. The LOD and LOQ for FB_2_ were 2.2 and 6.6 ng/g of sample, respectively. Concerning AFB_1_, the mean recovery was 82.1%, and the mean RSD was 8.8%. The respective LOD and LOQ for AFB_1_ were 0.3 ng/g and 0.9 ng/g.

**Figure 3:**
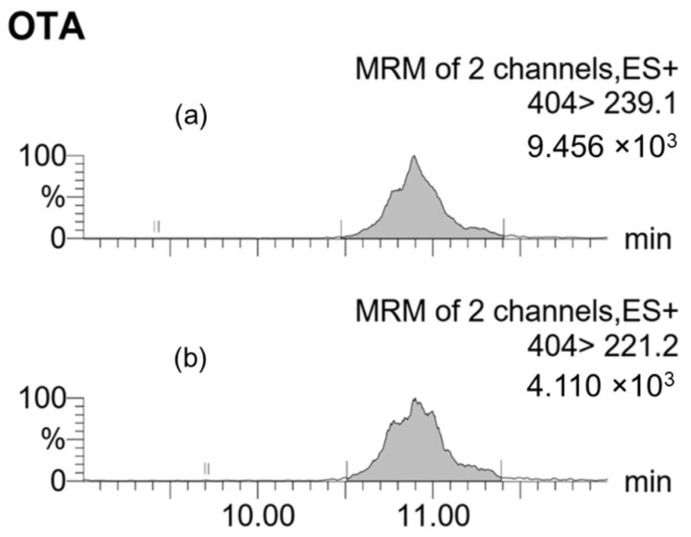
MRM chromatograms of a blank grape sample spiked with 10 ng/g of OTA extracted using the DLLME method. (**a**) Quantifier ion transition (q1: 404 > 239.1); (**b**) Qualifier ion transition (q2: 404 > 221.2). The shaded peaks correspond to OTA.

**Figure 4:**
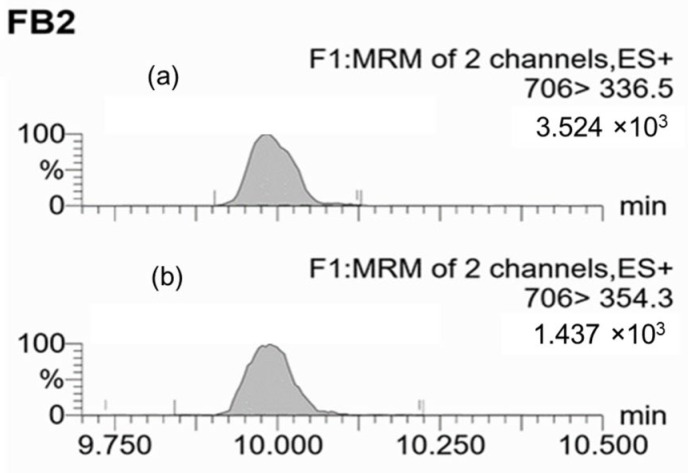
MRM chromatograms of a blank grape sample spiked with 10 ng of FB_2_/g. (**a**) Quantifier ion transition (q1: 706 > 336.5); (**b**) Qualifier ion transition (q2: 706 > 354.3). The shaded peaks correspond to FB_2_.

**Figure 5:**
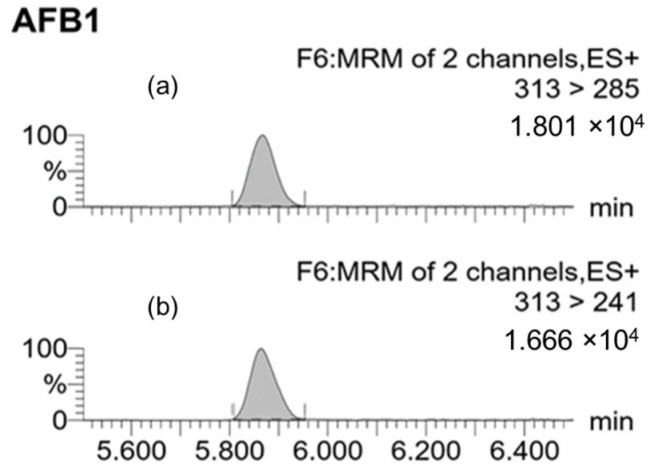
MRM chromatograms of a blank grape sample spiked with 20 ng of AFB_1_/g. (**a**) Quantifier ion transition (q1: 313 > 285); (**b**) Qualifier ion transition (q2: 313 > 241). The shaded peaks correspond to AFB_1_.

Multiple reaction monitoring (MRM) chromatograms were obtained by conducting UPLC−ESI(+)−MS/MS analysis of a blank grape sample spiked with a specific level of each toxin before extraction with the respective methods (Figure 3, Figure 4 and Figure 5). The chromatograms revealed the consistency of the retention times of both the q1 (quantifier) and q2 (qualifier) ions for each mycotoxin.

Based on UPLC-ESI-MS/MS analysis of all 85 samples obtained from six Spanish regions, OTA was detected in only 16 samples (19%). The mean concentration for OTA was 0.48 ± 0.10 ng/g with concentrations above the LOQ ranging from 0.38 ng/g to 0.7 ng/g. The highest concentration of OTA measured (a sample from the La Rioja region) was still three times lower than the regulated limit of 2.0 ng/g for wine and grape juice in the EU [4], and the FB_2_ and AFB_1_ mycotoxins were not detected in any of the analyzed samples.

We limited our PCR assessment of fungal presence to six mycotoxin-producing aspergilli. Of the 16 samples positive for OTA, five were contaminated exclusively with *A. niger,* representing La Rioja and Madrid regions. Three other samples showed co-contamination with multiple *Aspergillus* species: one sample showed the presence of *A. niger*, *A. carbonarius,* and *A. steynii* (Valencian Community), another sample contained *A. niger* and *A. westerdijkiae* (Madrid), and a third included genomic evidence of *A. westerdijkiae* and *A. steynii* (La Rioja). The remaining seven samples showed no presence of mycotoxin-producing aspergilli (see Supplementary Materials, Table S1).

## 3. Discussion

This study investigated the presence of six toxigenic *Aspergillus* species and their associated mycotoxins (OTA, FB_2_, and AFB_1_) in fresh grapes from organic vineyards located in Spanish regions with a strong viticultural tradition over three consecutive years. To the best of our knowledge, this is the first comprehensive survey of these mycotoxins in organic Spanish grapes before harvest.

Globally, organic viticulture has been increasing, with the organic vineyard area expanding by 13% per year, in contrast to a 0.4% decrease in conventional vineyard areas [34]. Organic practices prioritize natural and sustainable methods over the use of chemical products to maintain crop quality [35]. Previous studies have examined the impact of organic practices on the distribution of mycotoxin-producing black aspergilli in vineyards [32,33]. However, there is a current lack of data for the occurrence of key mycotoxin-producing fungi from other *Aspergillus* sections in organic vineyards.

In a previous metataxonomic study describing the mycobiota present in both conventional and organic vineyards, *A. flavus* was detected in nearly all grape samples, regardless of vineyard management practices, while *A. carbonarius* was absent [36]. That study was limited by a small sample size and highlighted the need for a more extensive investigation. In the present study, 85 samples were collected between 2019 and 2021 from various Spanish regions and subsequently analyzed. Despite the disparity of sample sizes from each region, which prevented the observation of regional trends, a similar pattern emerged across all samples: *A. flavus* was the predominant mycotoxin-producing species, while *A. carbonarius* was detected only occasionally. Consistent with previous findings, *A. niger* remained the most prevalent ochratoxigenic species, whereas fungi from the *Aspergillus* section *Circumdati* had a low occurrence [5,24,37,38].

The low incidence of *A. carbonarius* recovered in this study is consistent with other studies conducted in southern European countries. Testempasis et al. [32] found *A. carbonarius* in only 14% of Greek vineyard samples from 2018–2019. Similarly, Covarelli et al. [39] reported its absence in eight Italian vineyard samples collected between 2018 and 2019. A comparable trend was also observed in a similar recent study from Argentina, where *A. carbonarius* was not detected in any of the 24 grape samples collected from conventionally managed vineyards [40]. In contrast, studies on *Aspergillus* section *Flavi* colonizing grapes remain limited since *A. flavus* is not considered a serious risk in viticulture. However, EL Khoury et al. [24] reported a 43.1% prevalence of *A. flavus* in 470 grape samples from 27 distinct Lebanese vineyards, and Dutra-Silva et al. [41] observed a higher frequency of *Aspergillus* section *Flavi* than *Aspergillus* section *Nigri* in conventionally managed vineyards.

Although the present study focused on organic vineyards, the occurrence of *A. flavus* was comparable to that found in conventionally managed vineyards. Previous research suggests that farming practices may not significantly influence fungal diversity [42,43]. Palumbo et al. [44] found no significant differences in OTA-producing *Aspergillus* species between conventional and organic vineyards, and Lazzaro et al. [43] reported that annual climatic variations had a greater impact on fungal diversity in crops than the type of farming system. Therefore, the recent shift in the occurrence of toxigenic fungal populations in vineyards, observed in this study and previous reports [32,39,41], may be attributed to factors other than the type of farming practice (i.e., variations in climate conditions).

Temperature extremes and unpredictable weather patterns in southern Europe may favor certain fungal species while disadvantaging others [45,46,47]. According to recent modelling data, in southern and southeastern Europe, there is a high probability of both temperature increases and more frequent extreme weather events, which could influence fungal community dynamics [45]. For example, an increase in temperature occurred during the three years of grape sampling for this study, which was recorded by the Spanish National Weather Service (AEMET), whereby 2019–2021 were warmer than preceding years, with an increase in mean temperature of 0.5–1 °C [48,49,50].

*Aspergillus flavus*, in particular, is more virulent in warmer and drier climates. Battilani et al. [51] predicted increased AFB_1_ contamination in maize due to higher temperatures and drought conditions. Some authors, such as Paterson and Lima [25], have suggested that *A. flavus* might outcompete *A. carbonarius* and potentially other fungal species commonly found on grapes over the next century, as *A. flavus* infection is favored by rising temperatures and drier seasons compared to fungi like *A. carbonarius* [52,53,54,55,56]. Our findings support this trend, indicating a shift in the traditional balance of toxigenic fungi in vineyards, with *A. flavus* becoming increasingly prominent, while *A. carbonarius* may no longer represent a significant threat.

Regarding analytical methods, we employed dispersive liquid–liquid microextraction (DLLME) for the determination of OTA in grapes. Adjusting the pH to approximately 8 with NaHCO_3_ enhanced the efficacy of OTA extraction due to the solubility of its anionic forms. Subsequent acidification to a level below pH 3.4 retained most of the OTA in its neutral form, facilitating extraction by the numerous, minuscule droplets that formed when CHCl_3_ was combined with acetonitrile (ACN) to the filtered grape extract. This method proved superior to quick, easy, cheap, effective, rugged, and safe (QuEChERS) and liquid–liquid extraction (LLE) in terms of recovery rates and detection/quantification limits. Campone et al. [57] applied a similar pH shift to the DLLME approach when extracting mycotoxins from cereals, achieving recovery rates between 81.2% and 90.1%. Karami-Osboo et al. [58] used DLLME without a pH adjustment for extracting OTA from raisins, obtaining acceptable recoveries (68.6–85.2%). Ruan et al. [59] employed DLLME at pH 4 to extract OTA and citrinin from various fruits, incorporating NaCl to enhance extraction efficiency. In our method, NaCl was not added directly; however, it was generated in situ by the reaction of HCl with NaHCO_3_.

For the AFB_1_ extraction, we adapted the DLLME method applied to fruit juices by Pallarés et al. [60], adding the step of removing solid material from samples. We observed LOD and LOQ values of 0.3 and 0.9 ng/g, respectively, which are comparable to those reported by Pallarés et al. [60] for apple juice. Although the recoveries and RSDs in our spiked samples were satisfactory, AFB_1_ was not detected in any of the analyzed samples.

To extract FB_2_ from grapes, we modified the method employed by Logrieco et al. [17], which was applied to must samples, reducing both sample amount and solvent volumes and replacing rotary evaporation with air drying at 50 °C. The mean recovery was 79%, which is considered acceptable. Although FB_2_ was not detected in any of our samples, this mycotoxin has been previously detected in grapes, raisins, must, and wine, albeit at low levels (0.4–2.4 ng/mL) [17,18,19,61,62]. Notably, in another study that assessed FB_2_ contamination in grape samples, De Souza et al. [37] also failed to detect this toxin despite a high incidence of *A. niger*.

In our study, OTA was the only mycotoxin detected, with levels well below regulatory limits. OTA concentrations typically decrease during the winemaking process [63,64], suggesting that wines from Spanish organic grapes are unlikely to exceed the maximum permitted limit of 2 ng/mL. OTA was present in only 19% of samples, representing a lower incidence than previously reported in earlier studies [65,66]. García-Cela et al. [5] and De Souza et al. [37] also reported low OTA contamination despite high incidences of *A. niger* and *A. carbonarius*, which was mainly attributed to effective vineyard management practices.

The presence of mycotoxin-producing fungi on grapes is not an indication of mycotoxin contamination, as fungal spores may reside on the surface of grape berries without causing infection [67]. Most *Aspergilli* species are opportunistic pathogens that can only infect plants through an open wound or injury [68]. Moreover, mycotoxin production requires successful penetration of the berry tissue by the fungus [69]. Since 2009, integrated pest management (IPM) has improved vineyard practices across Europe, reducing mycotoxin risks through timely harvesting, proper pruning, and disease control [6,33,70,71]. Particularly, studies reporting high OTA incidence in vineyards were conducted before the widespread adoption of IPM [65,66].

In samples where OTA was detected, *A. niger* was the most frequently identified contaminant. A global increase in temperatures has altered the distribution of toxigenic fungi, increasing the prevalence of certain species in new environments or associated with new hosts [45,72,73]. Thus, OTA contamination in samples lacking known OTA producers may be attributed to uncommon or emerging species, highlighting the need for next-generation sequencing to better identify and monitor potential new threats.

AFB_1_ was not detected in any of our samples, despite a high occurrence of *A. flavus*. This result is consistent with previous studies that also failed to detect AFB_1_ in grapes or wine [74,75,76]. In the study by El Khoury et al. [24], which addressed both *A. flavus* occurrence and AFB_1_ contamination in grapes, only low levels of AFB_1_ were reported, close to our LOD. *A. flavus* isolates from grapes failed to produce AFB_1_ in grape-based media under various incubation conditions [77]. Moreover, as previously reported by other authors, any given *A. flavus* population also includes non-aflatoxigenic strains [78]. Nevertheless, the presence of *A. flavus* in vineyards should not be overlooked, because vineyards could serve as reservoirs for inoculum to spread to nearby fields with preferred hosts, such as barley, wheat, or oats, which are susceptible to *A. flavus* contamination [79,80,81]. Furthermore, *A. flavus* is known to produce other mycotoxins such as cyclopiazonic acid [82] or aflatrem [83], which, although not as serious as aflatoxin, still pose a health risk [84,85]. At present, the EU has not established maximum allowable limits for aflatoxins in wine or grape-derived products.

In conclusion, our findings indicate a significant shift in the landscape of mycotoxin-producing fungi across Spanish vineyards, possibly driven by fluctuating temperatures and weather patterns. *A. flavus* has become a more prevalent organism in vineyards, while *A. carbonarius* has declined. Although toxigenic fungi remain widespread, effective crop management can mitigate the risk of mycotoxin contamination. The increasing abundance of *A. flavus* poses potential risks to adjacent and preferred crop hosts, but only if the *A. flavus* strains present are aflatoxigenic, which warrants further investigation. The observed changes appear more closely linked to climate than organic farming practices, underscoring the need for additional studies in conventional vineyards to validate these trends. Future research could focus on the presence and impact of toxigenic fungi in crops cultivated near vineyards.

## 4. Materials and Methods

### 4.1. Collection of Grape Samples

**Figure 6:**
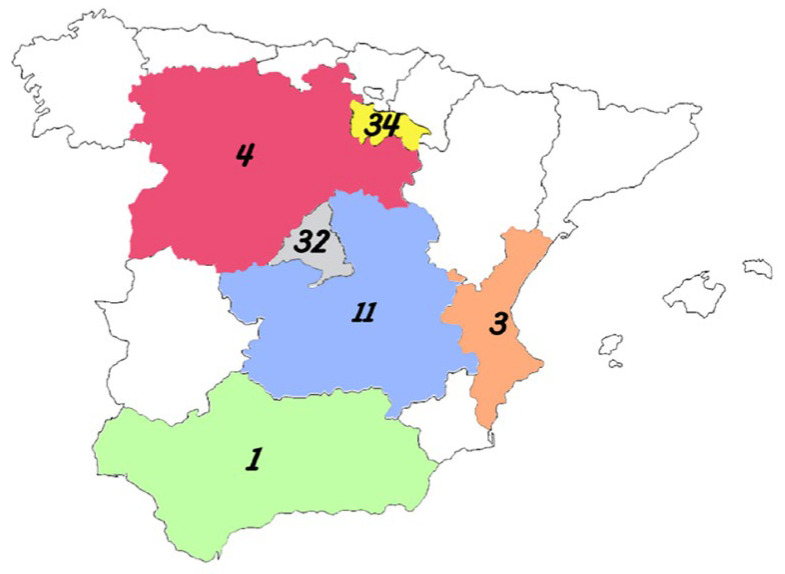
Map of Spain showing color-coded regions where organic grape samples were collected: Castilla and León (red), La Rioja (yellow), Madrid (gray), Castilla-La Mancha (blue), Valencian Community (orange), and Andalusia (green). Numbers in each region indicate the quantity of samples collected.

A total of 85 grape samples were collected at harvest time (mid-August through September) over three consecutive years (2019, 2020, and 2021) from organic vineyards located in six regions across Spain (Figure 6) with strong oenological traditions: Rioja (*n* = 34 samples), Castilla and Leon (*n* = 4), Madrid (*n* = 32), Castilla-La Mancha (*n* = 11), Valencian Community (*n* = 3), and Andalusia (*n* = 1). The regions selected for sampling were chosen based on their proximity to the authors’ laboratories, the high density of wineries in these areas, and existing collaborative agreements with local wine producers.

Sample plots ranging from 350 × 100 m^2^ to 500 × 100 m^2^ were randomly selected within each vineyard, from which 3 kg of grapes was collected. Sampling was conducted as follows: bunches were first collected from the grapevine closest to the center of the vineyard plot, followed by additional bunches collected 5 m away in each of the four cardinal directions from that central point, until a total weight of 3 kg was attained. Each 3 kg sample was destemmed, then its grapes were thoroughly mixed and subsequently divided into two 100 g batches. No distinction was made between intact/healthy or damaged/moldy grapes. All batches were initially stored at −20 °C. Batches undergoing molecular analysis were first superficially disinfected with 2.5% bleach and rinsed with distilled water for 2 min. They were then freeze-dried using a Cryodos freeze-dryer (Telstar, Madrid, Spain) for 48 h, sealed in plastic bags, and stored at 4 °C until they could be ground to a fine powder using a mortar and pestle and stored at −80 °C until ready for analysis. The grape batches intended for mycotoxin analysis were simply stored at 4 °C in sealed plastic bags, but they were allowed to reach room temperature before toxin analysis.

### 4.2. Occurrence of Toxigenic Aspergillus in Grape Samples

The presence of the most relevant toxigenic *Aspergillus* species was assessed using species-specific PCR protocols for *Aspergillus carbonarius* [86], *Aspergillus flavus* [87], *Aspergillus parasiticus* [88], *Aspergillus steynii*, and *Aspergillus westerdijkiae* [89]. Due to the evolving nature of the taxonomy of the *Aspergillus* section *Nigri*, particularly concerning the *A. niger* aggregate clade and the challenges in delineating species boundaries [90,91], we opted to consolidate the results using the most recent primers targeting ochratoxin-producing species previously grouped within this clade (*A. niger* and *A. welwitschiae*), as described by Palumbo and O’Keeffe [92]. For reporting purposes, these results are presented collectively under a single *A. niger* category. All species, except *A. carbonarius*, were identified using an initial species-specific PCR amplification following the protocols described by the aforementioned authors, and then by a reamplification step using 2 µL of the amplified products as a template, applying the same species-specific protocols. Amplification of *Aspergillus carbonarius* was performed via nested PCR with a first amplification of the ITS1-5.8S-ITS2 region using the protocol described by White et al. [93]. A second species-specific amplification was performed with 2 µL of the amplified products, according to the method described by Patiño et al. [86]. Primer sequences are provided in the Supplementary Materials (Table S2).

From each powdered grape batch, three 100 mg replicates were independently subjected to the extraction of total genomic DNA using the DNeasy Plant Mini Kit (Qiagen, Hilden, Germany). PCR amplification was performed using NZYTaq II 2x Green Master Mix (Nzytech, Lisbon, Portugal) with 8 µL of the extracted template. Amplified products were visualized by electrophoresis on 1.5% agarose gels (Pronadisa, Madrid, Spain) stained with 3 µL of Green Safe Premium (1 µg/mL) (NZYTech, Lisbon, Portugal) and 1 × TAE buffer (40 mM Tris-acetate and 1.0 mM EDTA). The amplicons were compared to NZYDNA Ladder V (NZYTech, Lisbon, Portugal), which was employed as a molecular size marker. After 25 min of electrophoresis at 80 V, visualization was carried out using a UV lamp (ETX-20-M, Vilber Lourmat, France). A sample was considered positive for the presence of a specific fungal species when at least two of the three replicates showed a specific amplification band.

### 4.3. Mycotoxin Analysis

#### 4.3.1. Reagents and Standards

Ethanol, methanol (MeOH), acetonitrile (ACN), formic acid, ethyl acetate, and chloroform (CHCl_3_) were purchased from Fluka (Merck, Darmstadt, Germany). Hydrochloric acid (HCl), sodium hydrogen carbonate (NaHCO_3_), sodium chloride (NaCl), and anhydrous magnesium sulfate (MgSO_4_) were purchased from Panreac (Merck). The C18 and mycotoxin standards of OTA (5 mg), FB_2_ (1 mg), and AFB_1_ (1 mg) were procured from Sigma-Aldrich (Merck). The OTA standard was dissolved in 5 mL of ethanol to provide a stock solution, while the FB_2_ and AFB_1_ standards were dissolved in 1 mL of ACN:water (1:1, *v*/*v*). Further diluted solutions were prepared with ACN:water (1:1, *v*/*v*) or MeOH:water (7:3, *v*/*v*) depending on the mycotoxin. All stock solutions were stored at −20 °C in total darkness to prevent quality degradation. The diluted standard solutions were used to prepare calibration curves and spiking experiments, kept at 4 °C, and monitored gravimetrically to avoid concentration changes. Water was purified using a Milli-Q apparatus (Millipore, Billerica, MA, USA).

#### 4.3.2. Assessment of Analytical Methods for OTA Detection

Three analytical methods were evaluated for their suitability in extracting and purifying OTA from grape samples. The first method, liquid–liquid extraction (LLE), was based on the protocol developed by De Jesus et al. [94]. The second method was based on the procedure of Wei et al. [95], which employed ACN and a QuEChERS (quick, easy, cheap, effective, rugged, and safe) approach for OTA extraction from grapes and other food matrices. The third method was adapted from Arroyo-Manzanares et al. [96], incorporating a pH shift as described by Campone et al. [57], and was originally developed for OTA determination in cereals using dispersive liquid–liquid microextraction (DLLME).

OTA exhibits both acidic and basic properties, with reported pKa values of 4.2–4.4 (pKa1) and 7.0–7.1 (pKa2) [97]. At pH values below 4.2, the neutral form of OTA predominates over the monoanionic species. Conversely, at pH values above 7.1, the dianionic form becomes dominant, increasing OTA’s solubility in aqueous media.

Details of the LLE and QuEChERS procedures are provided in the Supplementary Materials.

##### DLLME Method for OTA Analysis

Based on preliminary results, the DLLME method with a pH shift was identified as the most effective approach for extracting OTA from grapes. Five grams of homogenized grape berries was placed into a 50 mL Falcon tube, and 4 mL of 5% aqueous NaHCO_3_ solution was added. The tube was capped, vortexed for 5 min, and centrifuged at 10,000 rpm for 6 min. The supernatant was filtered through a 0.45 µm nylon syringe filter into a separate conical tube. The residue was washed with 2 mL of pure water, vortexed for 3 min, and centrifuged again under the same conditions. The resulting supernatant was filtered through a 0.45 µm nylon filter and combined with the previous filtrate. The combined extract was acidified to a pH slightly below 3 by adding drops of 1 M HCl to convert OTA into its neutral form. The solution was sonicated with short pulses to remove gas bubbles, primarily CO_2_, that had accumulated at the top of the solution. Subsequently, 1.6 mL of a solvent mixture consisting of CHCl_3_ (0.6 mL) and ACN (1 mL) was rapidly added to the tube. A cloudy emulsion formed, and the tube was vortexed for 1 min, followed by centrifugation at 10,000 rpm for 6 min. The organic phase was transferred to a clean tube. To recover any remaining OTA from the aqueous phase, 0.7 mL of CHCl_3_ was added, followed by vortexing for 1 min and centrifugation under the same conditions. The bottom organic phase was collected and combined with the previously separated organic extract. The combined organic phases were evaporated to dryness under a stream of nitrogen at 50 °C. The residue was reconstituted in 0.5 mL of ACN:water:formic acid (49.5:49.5:1, *v*/*v*/*v*), filtered through a 0.22 µm PTFE syringe filter, and transferred into a vial. An aliquot of the filtrate was used for UPLC–ESI–MS/MS analysis.

##### UPLC–ESI–MS/MS Analyses of Extracts from Organic Grapes

UPLC–ESI–MS/MS analysis of OTA, FB_2_, and AFB_1_ was performed using an ACQUITY UPLC™ system (Waters, Manchester, UK) coupled to an electrospray ionization interface operating in positive mode (ESI+), and connected to an ACQUITY TQD tandem quadrupole mass spectrometer (Waters Co., Milford, MA, USA). Chromatographic separation was achieved on a reversed-phase ACQUITY UPLC BEH C18 column (50 × 2.1 mm, 1.7 µm particle size; Waters). A 5 µL injection volume was used. The column temperature was maintained at 25 °C, and the autosampler was set to 15 °C.

The mobile phase consisted of (A) water containing ammonium formate (0.15 mmol/L) and formic acid (0.1%) and (B) MeOH at a flow rate of 0.35 mL/min using a gradient elution program [98]. Data acquisition and processing were carried out using MassLynx 4.1™ software (Waters, Manchester, UK).

Multiple reaction monitoring (MRM) chromatograms were acquired for each analyte. For each compound, the mean retention time (±2.5% tolerance) and the two most intense transitions from the ionized precursor to the corresponding product ions were monitored for identification. The most abundant transition (quantifier ion, q1) was used for quantification, while a second, less intense transition (qualifier ion, q2) was used for confirmation to enhance specificity. The q1/q2 ion ratio was also used as an additional confirmation criterion [99].

For OTA, the q1 and q2 transitions corresponded to the *m*/*z* 404.0 to 239.1 and 404.0 to 221.2, respectively. The retention time was 10.8 ± 0.2 min. The cone voltage and collision energy were 25 V and 20 eV for q1, and 40 V and 30 eV for q2, respectively.

##### Calibration and Validation

Matrix-matched calibration was employed for all mycotoxins analyzed in grape samples, as signal suppression or enhancement during the electrospray ionization (ESI) process has been reported for OTA, FB_2_, and AFB_1_ in fruit juice, grapes, and wine analyzed by UHPLC–ESI–MS/MS [60,76,95,100,101]. For OTA, a calibration curve was developed by injecting standard solutions into blank grape extracts processed according to the protocol for the DLLME procedure, as described in the section titled DLLME Method for OTA Analysis. In the case of the alternative extraction methods (LLE and QuEChERS), the calibration curves were constructed similarly, following the procedures described in the Supplementary Materials. After final evaporation to dryness, the residues were spiked with OTA standard solutions and brought to 1.0 mL with ACN:water:formic acid (49.5:49.5:1, *v*/*v*/*v*). Calibration standards at different OTA concentrations in the grape matrix were then injected into the UPLC–ESI–MS/MS system. A weighted least squares (WLS) regression using 1/*x* as the weighting factor, where *x* is the concentration of the standard, was used to calculate the parameters of the calibration curve for OTA, FB_2,_ and AFB_1_. For all mycotoxins, the limit of detection (LOD) was estimated to be 3.3 × *s*/*b*, where *s* is the standard error of the estimate and *b* is the slope of the calibration line generated by using blank samples containing standards at low levels, close to the limit of quantification (LOQ), which was estimated to be 3 × LOD [102].

The three analytical methods for OTA (LLE, QuEChERS, and DLLME) were compared in terms of recovery percentage and relative standard deviation (RSD). For each method, homogenized blank grape samples were spiked in triplicate with OTA at four concentrations (2, 4, 10, and 20 ng/g). The spiked samples were shaken and left uncovered at room temperature for 2 h to allow partial solvent evaporation before being processed and analyzed as described for grape samples in the appropriate protocols (see Supplementary Materials for LLE and QuEChERS).

#### 4.3.3. Analytical Method for FB_2_

A method based on that described in Logrieco et al. [17] was used. Five grams of homogenized berries was weighed into a 50 mL Falcon tube, and fifteen mL of ethyl acetate was added. The tube was shaken for 30 min using a rotary shaker (model F-205, FALC Instruments S.R.L.; Treviglio, Italy). The mixture was centrifuged at 5000 rpm for 10 min. The supernatant was transferred to a clean tube, and the residue was re-extracted with an additional 15 mL of ethyl acetate following the same procedure. The two extracts were combined and filtered through 110 mm filter paper (Whatman, Maidstone, UK). The filtrate was evaporated under a stream of air at 50 °C. The residue was dissolved in 2 mL of a MeOH:water solution (7:3, *v*/*v*) by vortexing for 2–3 min. The solution was then filtered through a 0.22 µm nylon syringe filter into a vial for chromatographic analysis.

For FB_2_ determination, the q1 and q2 transitions corresponded to *m*/*z* 706 to 336.5 and 706 to 354.3, respectively. The cone voltage was set at 50 V and the collision energy at 40 eV for both transitions. The retention time was 10.0 ± 0.2 min.

For matrix-matched calibration, a blank grape sample was extracted repeatedly. The resulting evaporated residues were spiked with various volumes of FB_2_ standard solutions prepared in MeOH:water (7:3, *v*/*v*). Final volumes were adjusted to 2 mL with the solvent and injected into the UPLC–ESI–MS/MS instrument. The FB_2_ concentrations in the calibration solutions were 10, 25, 50, 100, 200, and 500 ng/mL. Method validation was performed by spiking blank grape samples with FB_2_ at four concentrations: 10, 20, 50, and 150 ng/g. Each level was analyzed in five replicate injections.

#### 4.3.4. Analytical Method for AFB_1_

The method described in Pallarés et al. [60] for fruit juices was adapted for the extraction of AFB_1_ from grapes. Five grams of homogenized berries was mixed with 4 mL of pure water in a 15 mL Falcon tube, vortexed for 5 min, and centrifuged at 10,000 rpm for 6 min. The supernatant was filtered through a 0.45 µm nylon syringe filter into a 15 mL conical tube. The pellet was washed with 2 mL of pure water, vortexed for 2 min, and centrifuged again under the same conditions. The resulting supernatant was filtered through a 0.45 µm nylon syringe filter and combined with the previous filtrate. One gram of NaCl was added. The mixture was vortexed for 2–3 min. A two-step DLLME procedure was applied. In the first step, a mixture of disperser solvent (0.950 mL ACN) and extraction solvent (0.620 mL ethyl acetate) was rapidly added with a syringe. The tube was vortexed for 1 min, forming a cloudy solution. This mixture was centrifuged at 5000 rpm for 5 min, and 600 µL of the upper organic phase was collected with a syringe and transferred to another conical tube. In the second step, a mixture of disperser solvent (0.950 mL of MeOH) and extraction solvent (0.620 mL of CHCl_3_) was quickly added to the remaining aqueous phase. After centrifugation at 5000 rpm for 10 min, 600 µL of the bottom organic phase was collected and combined with the previously obtained organic phase. The combined organic extracts were evaporated to near dryness under a N_2_ stream at 50 °C. The residue was reconstituted in 1 mL of ACN:MeOH (50:50, *v*/*v*) and filtered through a 0.22 µm nylon syringe filter into a vial for chromatographic analysis.

The q1 and the q2 transitions correspond to *m*/*z* 313 to 285 and 313 to 241, respectively. The cone voltage was set at 70 V for both transitions, with collision energies of 25 eV (q1) and 35 eV (q2). The retention time was 5.9 ± 0.1 min.

For calibration, a blank grape sample was extracted, and standard solutions of AFB_1_ in ACN:MeOH (50:50, *v*/*v*) were added to the dry residues. After vortexing, the solutions were filtered through a 0.22 µm nylon filter into vials for chromatographic analysis. The calibration curve was prepared using AFB_1_ standard concentrations of 1, 2, 5, 10, 20, 50, and 100 ng/mL. Method validation was performed by spiking blank grape samples with AFB_1_ at four concentration levels (1, 5, 10, and 20 ng/g). Each level was analyzed in five replicate injections.

## Figures and Tables

**Table 1:** Average recoveries (%) and relative standard deviations (*n* = 5) of OTA when spiked into blank grape samples at four concentrations, based on three different extraction methods.

	Extraction/Cleanup Method		
Spiked OTA (ng/g) in Grape Berries	DLLME	LLE	QuEChERS
2	70 ± 11	29 ± 10	46 ± 13
4	79 ± 8	35 ± 11	56 ± 9
10	90 ± 7	44 ± 12	65 ± 10
20	93 ± 6	55 ± 10	76 ± 6

DLLME: dispersive liquid–liquid microextraction; LLE: liquid–liquid extraction; QuEChERS: quick, easy, cheap, effective, rugged, and safe.

**Table 2:** Comparison of validation parameters for the determination of fumonisin B_2_ and aflatoxin B_1_ concentrations from organic grape samples.

Mycotoxin	Spiking Level (ng/g)	Recovery (%)	RSD of Recovery (%)	LOD (ng/g)	LOQ (ng/g)
Fumonisin B_2_	10	70.3	16.2	2.2	6.6
	20	75.2	11.6		
	50	80.1	8.4		
	150	90.7	6.2		
Aflatoxin B_1_	1	73.2	13.4	0.3	0.9
	5	77.9	9.8		
	10	84.8	6.8		
	20	92.3	5.2		

LOD: limit of detection; LOQ: limit of quantification.

## Data Availability

Data is available in the Article and the Supplementary Materials.

## Supplementary Materials

The following supporting information can be downloaded at: https://www.mdpi.com/article/10.3390/toxins17100487/s1, Tables S1 and S2; Alternative methods for OTA extraction and determination: LLE and QuEChERS.
