# Supplementary material for: Toxigenic Aspergillus Diversity and Mycotoxins in Organic Spanish Grape Berries
Source: Toxins (Basel). 2025 Sep 30;17(10):487. doi: 10.3390/toxins17100487 (PMC12567864; doi:10.3390/toxins17100487)
Supplement: Supplementary file 1 [file toxins-17-00487-s001.zip › toxins-3843467-supplementary.pdf]

Supplementary Materials for the MS Toxins-3843467

Title: Toxigenic *Aspergillus* Diversity and Mycotoxins in Organic Spanish Grape Berries

Table S1. Summary of the toxigenic *Aspergillus* species detected in each of the grape samples tested, as well as the levels of ochratoxin A (OTA) in the samples. Contamination by fumonisin B_2 and aflatoxin B_1 was also analyzed; however, none of the samples tested positive for either toxin.

			Toxigenic *Aspergillus*					
**Sample ID**	**Region**	**Concentration of OTA (ng/g)**	***A. flavus***	***A. parasiticus***	***A. carbonarius***	***A. steynii***	***A. westerdijkiae***	***A. niger***
1	Madrid	ND	+	-	-	+	-	+
2	Madrid	ND	+	-	-	-	-	-
3	Valencia	ND	+	-	-	-	-	+
4	Castilla and Leon	ND	+	-	-	-	-	-
5	Castilla and Leon	ND	+	-	-	-	-	+
6	Castilla and Leon	ND	+	-	-	-	-	+
7	Madrid	ND	+	-	+	-	-	+
8	Castilla La Mancha	ND	+	-	+	-	-	+
9	Madrid	ND	-	-	-	-	-	-
10	Madrid	ND	+	-	+	-	-	-
11	Madrid	ND	-	-	-	-	-	-
12	Madrid	ND	-	-	+	-	-	-
13	Valencia	ND	+	-	-	-	-	-
14	La Rioja	ND	-	-	-	-	-	+
15	Castilla La Mancha	ND	+	-	-	-	-	-
16	Castilla La Mancha	ND	-	-	-	-	-	+
17	Madrid	ND	+	-	-	-	-	+
18	Madrid	ND	+	-	+	-	-	-
19	Madrid	ND	+	-	-	-	+	+
20	Castilla and Leon	ND	-	-	-	-	-	+
21	Castilla La Mancha	ND	-	-	-	-	-	+
22	Castilla La Mancha	ND	-	-	-	-	-	+
23	Castilla La Mancha	ND	-	-	-	-	-	+
24	Madrid	ND	-	-	-	-	-	-
25	Madrid	ND	+	-	-	-	+	-
26	Madrid	ND	+	-	-	-	+	-
27	Madrid	ND	-	-	-	-	-	-
28	Madrid	0.45	-	-	-	-	-	-
29	Madrid	ND	+	-	-	-	+	-
30	Madrid	ND	-	-	-	-	-	-
31	Madrid	ND	-	-	-	-	-	-
32	Madrid	0.6	-	-	-	-	-	-
33	Madrid	D	+	-	-	-	-	+
34	Madrid	ND	-	-	-	-	-	-
35	Castilla La Mancha	ND	+	-	-	-	-	-
36	Castilla La Mancha	ND	+	+	+	-	-	+
37	Castilla La Mancha	ND	+	+	-	-	-	+
38	Madrid	ND	+	-	-	-	-	-

39	Madrid	ND	+	+	-	-	-	-
40	Madrid	0.38	+	-	-	-	-	-
41	Madrid	ND	+	-	+	-	+	+
42	Madrid	ND	+	+	-	-	-	+
43	Madrid	D	+	-	-	-	+	+
44	Madrid	ND	+	-	-	-	-	-
45	Madrid	ND	-	-	-	-	-	-
46	Madrid	ND	+	+	-	-	-	+
47	Castilla La Mancha	ND	+	+	+	-	-	+
48	Castilla La Mancha	ND	-	-	-	-	-	-
49	Valencia	D	+	+	+	+	-	+
50	Madrid	0.40	-	-	-	-	-	-
51	Andalucia	0.46	-	-	-	-	-	-
52	Madrid	ND	+	+	+	+	-	+
53	La Rioja	ND	-	-	-	-	-	-
54	La Rioja	ND	+	+	-	+	+	-
55	La Rioja	ND	+	+	-	+	+	+
56	La Rioja	ND	+	-	-	+	-	-
57	La Rioja	0.4	+	-	-	-	-	+
58	La Rioja	ND	+	-	-	-	-	-
59	La Rioja	ND	-	-	-	+	-	+
60	La Rioja	ND	+	-	-	-	-	-
61	La Rioja	ND	+	+	-	+	-	+
62	La Rioja	ND	+	-	-	-	-	-
63	La Rioja	0.5	-	-	-	-	-	-
64	La Rioja	D	+	+	-	-	-	+
65	La Rioja	ND	+	-	-	-	+	+
66	La Rioja	ND	+	-	+	-	-	+
67	La Rioja	ND	-	-	-	-	-	-
68	La Rioja	ND	+	+	-	-	-	+
69	La Rioja	ND	+	-	-	-	-	-
70	La Rioja	0.7	+	-	+	+	-	+
71	La Rioja	0.45	+	-	-	+	+	-
72	La Rioja	D	+	+	-	-	-	+
73	La Rioja	0.6	-	+	-	-	-	-
74	La Rioja	ND	+	+	-	-	-	+
75	La Rioja	ND	+	-	-	-	-	-
76	La Rioja	ND	+	-	-	+	-	-
77	La Rioja	ND	+	-	-	+	-	-
78	La Rioja	ND	+	-	-	-	-	-
79	La Rioja	ND	+	-	-	-	-	-
80	La Rioja	0.38	+	+	-	-	-	+
81	La Rioja	ND	+	-	-	-	-	-
82	La Rioja	ND	-	-	-	-	-	+
83	La Rioja	ND	+	-	-	-	-	-
84	La Rioja	ND	-	-	-	-	-	-
85	La Rioja	ND	-	-	-	-	-	-

ND: OTA not detected; D: OTA detected at levels between the LOD and the LOQ; +: detected; -: not detected.

Table S2. Specific primers used in this study

Aspergillus species detected	Primer ID	Sequence	PCR Conditions	Reference
Aspergillus carbonarius	CAR1	5′ GCATCTCTGCCCCTCGG 3′	1 cycle of 4 min 30 s at 95 °C, 25 cycles of 30 s at 95 °C, 25 s at 59 °C, 40 s at 72 °C and 1 cycle of 5 min at 72 °C	[86]
	CAR2	5′ GGTTGGAGTTGTCGGCAG 3′		
Aspergillus flavus	FLA1	5′ GTAGGGTTCCTAGCGAGCC 3′	1 cycle of 5 min at 95 °C, 26 cycles of 30s at 95 °C, 30 s at 58 °C, 45 s at 72 °C and 1 cycle of 5 min at 72 °C	[87]
	FLA2	5′ GGAAAAAGATTGATTTGCGTTC 3′		
Aspergillus parasiticus	PAR1	5′ GTCATGGCCGCCGGGGGCGTC 3′	1 cycle of 5 min at 95 °C, 26 cycles of 30 s at 95 °C, 30 s at 69.3 °C, 30 s at 72 °C and 1 cycle of 5 min at 72 °C	[88]
	PAR2	5′ CCTGGAAAAAATGGTTGTTTTGCG 3′		
Aspergillus steynii	STEYF	5′ CCACGCGGCCGCCGGGGGAG 3′	5 min at 95 °C, 21 cycles of 30 s at 95 °C, 45 s at 72 °C and 5 min at 72 °C	[89]
	STEYR	5′ CGGGGGGGACGAGGACCCAAC 3′		
Aspergillus westerdijkiae	WESTF	5′ CTTCCTTAGGGGTGGCACAG 3′	5 min at 95 °C, 22 cycles of 30 s at 95 °C, 30 s at 63°C, and 40 s at 72°C and 5 min at 72 °C	
	WESTR	5′ CAACCTGATGAAATAGATTGGTTG 3′		
Aspergillus niger	AnF	5' GGATTTCGACAGCATTTTCCAGAACG 3'	5 min at 95 °C, 35 cycles of 30 s at 95 °C, 30 s at 66 °C, and 30 s at 72 °C, with a final extension of 5 min at 72 °C	[92]
	AnR	5' GATAAAACCATTGTTGTCGCGGTCG 3'		
	AwF	5' GGGATTTCGACAGCATTTCTCAGAATT 3'		
	AwR	5' GATAAAACCATTGTTGTCGCGGTCA 3'		

Alternative Methods for OTA Extraction and Cleanup Evaluated in Organic Grape Samples (Other than DLLME).

1. Liquid–Liquid Extraction (LLE) Method

Half a gram of homogenized berries was placed into a 2 mL Eppendorf tube and extracted with 1 mL of chloroform ($CHCl_3$) by vortexing for 5 minutes. The tube was then centrifuged at 10,000 rpm for 5 minutes. The upper aqueous phase, along with the interphase containing skins and seeds, was carefully transferred to another tube. The bottom organic phase was collected and transferred to an amber glass vial. A second extraction was performed by adding 1 mL of $CHCl_3$ to the remaining pellet, followed by vortexing for 5 minutes and centrifugation under the same conditions. The resulting organic phase was combined with the first extract in the amber vial. The solvent was evaporated under a gentle stream of N_2 at 50 °C. The residue was reconstituted in

0.3 mL of acetonitrile:water:formic acid (49.5:49.5:1, v/v/v), vortexed briefly, and filtered through a 0.22 μm PTFE syringe filter. The solution was then analyzed by UPLC–ESI–MS/MS.

2. QuEChERS (Quick, Easy, Cheap, Effective, Rugged, and Safe) Method

Ten grams of homogenized berries was placed into a 50 mL Falcon tube and extracted with 10 mL of acetonitrile (ACN) by shaking vigorously for 10 minutes. One gram of sodium chloride (NaCl) and 4 g of anhydrous magnesium sulfate ($MgSO_4$) were then added. The tube was vortexed for 5 minutes and centrifuged at 5,000 rpm for 5 minutes. A 1.5 mL aliquot of the supernatant was transferred to a 2 mL Eppendorf tube containing 50 mg of C18 and 150 mg of anhydrous $MgSO_4$. The tube was vortexed for 1 minute and centrifuged at 10,000 rpm for 5 minutes. The upper phase was filtered through a 0.45 μm nylon syringe filter into a vial. The solvent was evaporated under a gentle stream of N_2 at 50 °C. The residue was reconstituted in 1.5 mL of acetonitrile:water:formic acid (49.5:49.5:1, v/v/v), and the solution was analyzed by UPLC–ESI–MS/MS.
